# A comparative study between two methods of delivery of chemotherapeutic agent in patients with bone and soft tissue sarcoma of lower extremity

**DOI:** 10.1186/s12891-023-06417-7

**Published:** 2023-04-22

**Authors:** Jing Shan, Sumei Lv, Haihong Li, Donglai Wang, Xiaoyu Zhang, Wei Liu

**Affiliations:** 1 Department of Orthopedics, The Fourth Hospital of Hebei Medical University, Hebei Provincial Cancer Institute, Shijiazhuang, Hebei China; 2 Department of Orthopedics, Cangzhou Central Hospital, Cangzhou, Hebei China

**Keywords:** Shoulder function, Chemotherapy, Catheter complications

## Abstract

**Background:**

We aimed to compare the effects of peripherally inserted central catheters (PICC) and implantable venous access devices (TIVADs) in terms of complications and shoulder function in patients with malignant bone and soft tissue tumors of the lower extremities.

**Methods:**

We analyzed 65 cases of TIVADs (chest wall) and 65 cases of PICC at the orthopedic department of the Fourth Hospital of Hebei Medical University between June 2019 and December 2021, which were diagnosed with malignant bone tumors or soft tissue tumors of the lower extremities (tumors had to be relatively sensitive to chemotherapy), received regular chemotherapy, with ≥ 14 cycles (42 weeks). The two groups were compared in terms of catheter indwelling time, catheter-related complications, Constant-Murley shoulder function score, and displacement of the position of the catheter end on the catheterization side.

**Results:**

Compared to the PICC group, at six months after catheterization, the TIVADs group reported better outcomes for catheter indwelling time, catheter-related complications, and Constant-Murley score for the catheterization-side shoulder joint (p < 0.05). The TIVADs group also reported less displacement of the catheter end position after 180° abduction of the catheterization-side shoulder joint (p < 0.05).

**Conclusions:**

Compared with PICC, TIVADs can prolong catheter indwelling time, reduce catheter-related complications, and maintain shoulder joint function, which makes it an ideal venous-access approach when providing chemotherapy to patients with malignant bone and soft tissue tumors of the lower extremities.

## Background

Malignant bone and soft tissue tumors of the lower extremities represent a disease that can seriously endanger human health; clinically, such tumors can be divided into malignant bone tumors and soft tissue sarcomas, respectively. The most common malignant bone tumors include osteosarcoma and Ewing sarcoma, and the most common soft tissue sarcomas include synovial sarcoma, undifferentiated sarcoma, and liposarcoma [1]. The 2016 annual report of China’s National Central Cancer Registry showed that in China, the incidence of primary malignant bone tumors is 1.44 per 100,000 people, and that such tumors account for 0.7% of all malignant tumors among patients [2]. Before the widespread use of chemotherapy, the long-term survival rate of patients with osteosarcoma was only 20–40%, and that of STS was only 35%. The five-year survival rate for patients treated with chemotherapy and surgical excision is 60–80% [3]. The primary treatments are amputation or extensive local excision and adequate radiotherapy, which often result in permanent disability, a survival rate of less than 20%, and a high local recurrence rate [4]. Currently, surgical treatment is the primary treatment for bone and soft tissue sarcoma. Neoadjuvant chemotherapy is performed prior to other treatments such as surgery in order to conduct early systemic treatment, eliminate potential micro-lesions, and improve the success rate of limb salvage surgery for postoperative chemotherapy and tumor reduction. The clinical application of first-line chemotherapy drugs include methotrexate, liposomal Adriamycin, and cisplatin. In spite of this, more than 50% of patients still have distant metastasis, and the toxic and side effects of chemotherapy drugs are evident, which has become one of the most important factors limiting its efficacy [5]. Currently, immunotherapy is a contentious problem in the treatment of bone and soft tissue tumors, which relies on stimulating the host immune system’s natural defense to attack and kill malignant cells. Immunotherapy’s mechanism differs from that of chemotherapy [6]. Compared with traditional treatment methods, such as surgery, radiotherapy, and chemotherapy, immunotherapy is more effective in curing the root cause and exhibits high selectivity, selectively killing malignant cells and reducing the damage to normal tissues [7, 8]. However, it is not mature at present and must be combined with surgery, radiotherapy, chemotherapy, and other treatment approaches.

Surgical treatment predominantly includes extensive and radical excision, both of which can cause significant bone defects, causing serious limb dysfunction and potential loss. Studies have demonstrated that only about 13–14% of patients with surgical treatment exhibit no impact on their physical functions [9]. Most patients experience varying degrees of lower limb mobility disorders, and a small number of amputees need to walk and live with the aid of walkers or axillary poles. Daily activities require upper limb assistance, which significantly impacts their quality of life. Moreover, patients often need to be combined with longer cycles of chemotherapy to maintain treatment. Owing to the short interval of chemotherapy and application of large doses of chemotherapy drugs, central vein access is essential for continuing patients’ chemotherapy. Currently, a PICC catheter is implanted in the upper limb, which affects patients’ walking with the aid of walker or axillary rod [10], and also affects patients’ functional exercise and quality of life [11–13]. The PICC catheter cannot be maintained for a long time, and needs to be re-punctured in each treatment cycle, thus increasing the total cost of treatment. As patients inevitably need to walk with a walker, the incidence of catheter complications, such as tube blockage and bleeding, is increased [14].

In 1982, the MD Anderson Cancer Center in the United States became the first institution to apply implantable central venous access devices through the cephalic vein in patients with tumors who were receiving chemotherapy [15]. This approach was then generalized and used worldwide, and gradually became the first-choice approach for infusion devices that can be indwelled in the body for a long time and buried in the subcutaneous tissue [16]. Today, this approach is widely used in patients undergoing chemotherapy.

To understand the advantages and disadvantages of the two catheterization methods (PICC and TIVADs) and help clinical workers make better choices, this study compares their effects in the post-catheterization period.

## Methods

This study was approved by the Ethics Committee of the Fourth Hospital of Hebei Medical University, and written informed consent was acquired from the patients. The participants were informed about the study, including any benefits and risks involved, and the current study conformed to the principles of the Declaration of Helsinki.

### Sample and setting

We conducted a retrospective analysis of 65 patients who were administered an implantable venous access device (TIVADs) between June 2019 and December 2021, and 65 patients who were administered a PICC from June 2019 to December 2021 at the orthopedics department of the Fourth Hospital of Hebei Medical University. Before data collection, we consulted relevant literature and calculated the sample size of the two groups. In the process of data review, there was no loss of follow-up or missing data in the two groups. All of the patients had malignant bone and soft tissue tumors of the lower extremities, completed surgery (surgical types: extensive surgical resection or radical resection of the tumor, and new adjuvant chemotherapy before surgery) and had received chemotherapy [according to different types of tumor chemotherapy options: MAP regimen (high-dose methotrexate, doxorubicin, cisplatin); AP regimen (doxorubicin, cisplatin); VAC regimen (vincristine, doxorubicin, cyclophosphamide)].

#### Inclusion criteria

- Diagnosed with malignant bone tumors or soft tissue tumors of the lower (and the tumors are relatively sensitive to chemotherapy).
- Receiving regular chemotherapy, with ≥ 14 cycles (42 weeks) for malignant bone tumors and ≥ 6 cycles (24 weeks) for soft tissue sarcomas [17].
- No catheter-related contraindications.
- Good compliance.

#### Exclusion criteria

- Patients who could not complete chemotherapy or ceased treatment because of serious complications during chemotherapy.
- Patients who experienced complications during surgery that affected their chemotherapy cycles.

### Instruments

#### Constant-murley score

The Constant-Murley Score (CMS) [18] evaluates shoulder joint function, and contains four subscales: pain, activities of daily living, range of motion, and strength. Maximum scores for these subscales are 15 points for the pain scale, 20 points for the activities of daily living scale, 40 points for the range of motion scale, and 25 points for the strength scale. Higher scores indicate better shoulder joint function. Pain and activities of daily living are self-reported by the patients based on consideration of their actual status in this regard, and scores for range of motion and strength are given by medical staff after assessment.

#### Eastern Cooperative Oncology Group - Performance Status Rating

The Eastern Cooperative Oncology Group - Performance Status Rating (ECOG-PSR) [19] scale was used to evaluate the patients’ general physical condition and level of activity. This scale is scored by the patients’ caregivers, and scores range from 0 to 5 (0 = able to perform normal activities; 1 = symptomatic, but capable of performing almost all self-care activities; 2 = sometimes bedbound; in bed for < 50% of the day; 3 = bedbound; in bed for > 50% of the day; 4 = totally confined to bed; 5 = deceased).

#### Venous Port and PICC package

The PICC group used the three-way valve puncture package produced by Bard (Salt Lake City, USA). Meanwhile, the TIVADs group used the Bard Port implantation pump and the Groshong three-way valve infusion catheter (also produced by Bard).

#### Venous port implantation

Venous port implantation was usually performed through the internal jugular vein or subclavian vein. The catheter eventually entered the superior vena cava, and the ideal position for catheter end was the junction between superior vena cava and right atrium. After the catheter was placed, a leather bag was set up at the subclavian fossa in order to fix the infusion set; the subcutaneous tunnel was also set up, and the thickness of the subcutaneous tissue embedding the infusion set was ideally 0.5–1.0 cm. The noninvasive needle penetrated the bottom of the TIVADs vertically through the skin, and then, it was connected to the infusion device for use.

#### Catheterization of PICC group

The puncture site was 2-fifinger under the elbow fossa or above the elbow. The preferred needle insertion vein is basilic vein, followed by the median cubital vein, and then the cephalic vein. The catheter was inserted into the superior vena cava via basilic vein, and the catheter insertion length is from the puncture point to the left and right sternoclavicular joints along the vein, plus the section down to the third intercostal space. In general, the catheter insertion length was 40–55 cm. After insertion, the catheter was covered and fixed with a sterile transparent film. After catheterization, the position of the catheter end was confirmed by X-ray examination.

### Variables

Comparison of the two groups (PICC and TIVADs) in terms of disease type, gender, age, and general condition before chemotherapy showed no significant differences (Table 1). Observation measures included catheter indwelling time; catheter-related complications (phlebitis, catheter-related infection, catheter occlusion, catheter fracture/displacement, etc.); CMS score for the catheterization side at one month after catheterization, six months after catheterization, and at three months after extubation; and X-ray measurement of the displacement of the position of the catheter end after 180° abduction of the shoulder from the neutral position.

**Table 1:** Comparison of the Two Groups in terms of General Data

		PICC (n = 65)	TIVADs (n = 65)	t/χ^2^	*P*
Diseases	Bone tumor Soft tissue sarcoma	35 30	30 35	0.063	0.831
Gender	Female Male	25 40	37 28	0.077	0.912
Pre-Chemotherapy ECOG-PSR Score	0 1 2	15 42 8	12 43 10	− 0.39	0.851
Age (years)		41.22 ± 3.10	39.91 ± 3.22	0.287	0.776

ECOG-PSR: Eastern Cooperative Oncology Group - Performance Status Rating; PICC: peripherally inserted central catheter; TIVADs: implantable venous access device

### Statistical analyses

IBM SPSS Statistics for Windows, version 23.0 (IBM Corp, Armonk, NY, USA), was used for statistical processing. Independent samples t-tests were used to perform comparisons between the groups, while χ^2^ tests were used for comparisons of rates (phlebitis, catheter-related infection, catheter occlusion, catheter fracture/displacement, etc.) between the two groups. Analysis of variance was used for pairwise comparison of CMS scores for the catheterization side at one month after catheterization, at six months after catheterization, and at three months after extubation. Regarding the test level, α = 0.05; meanwhile, P < 0.05 was considered to indicate significant statistical difference.

## Results

### General data

In the PICC group, there were 40 males and 25 females, and the mean age was 41.22 ± 3.10 years. This group included 35 patients with malignant bone tumors (22 with osteosarcoma and 13 with Ewing sarcoma) and 30 patients with soft tissue sarcomas (12 with undifferentiated sarcoma, eight with synovial sarcoma, five with myxosarcoma, three with epithelioid sarcoma, and two with rhabdomyosarcoma).

In the TIVADs group, there were 28 males and 37 females, and the mean age was 39.91 ± 3.22 years. This group included 30 patients with malignant bone tumors (18 with osteosarcoma and 12 with Ewing sarcoma) and 35 patients with soft tissue sarcomas (12 with synovial sarcoma, 11with undifferentiated sarcoma, eight with myxoliposarcoma, two with rhabdomyosarcoma, and two with myxofibroblastic sarcoma).

### ECOG-PSR score before chemotherapy

For the PICC group, the pre-chemotherapy ECOG-PSR scores were as follows: 15 patients scored 0, 42 patients scored 1, and eight patients scored 2. For the TIVADs group, these scores were as follows: 12 patients scored 0, 43 patients scored 1, and 10 patients scored 2. There was no significant difference between the two groups regarding age, gender, disease type, or pre-chemotherapy ECOG-PSR score (Table 1).

### Catheter indwelling time

All patients in the PICC group were extubated within two weeks of completion of chemotherapy. In the TIVADs group, the port was removed once no abnormality was found at a re-examination conducted three months after the completion of chemotherapy. In the PICC group, the mean extubation time was 233.35 ± 10.43 days for patients with soft tissue sarcoma and 349.54 ± 15.08 days for those with malignant bone tumors, while in the TIVADs group the mean extubation time was 323.90 ± 7.77 days for patients with soft tissue sarcoma and 450.13 ± 13.56 days for those with malignant bone tumors (Table 2).

**Table 2:** Comparison of the Two Groups in terms of Catheter Indwelling Time

		PICC (n = 65)	TIVADs (n = 65)	T	*P*
Indwelling time (days)	Bone tumor Soft tissue sarcoma	349.54 ± 15.08 233.35 ± 10.43	450.13 ± 13.56 323.90 ± 7.77	−19.54 −30.62	0.000 0.000

PICC: peripherally inserted central catheter; TIVADs: implantable venous access device

### Constant-murley shoulder function score for the catheterization side

At one-month post-catheterization, the average CMS score for the PICC group was 94.00 ± 2.47 points, and that for the TIVADs group was 94.37 ± 3.01 points; statistical analysis revealed no significant difference between the two groups in this regard. At six months post-catheterization, the average score for the PICC group was 90.71 ± 2.81 points, and that for the TIVADs group was 94.20 ± 3.20 points; there was a significant difference (P = 0.034)between the two groups in this regard. At three months after extubation, the average score for the PICC group was 93.66 ± 2.27 points, and that for the TIVADs group was 94.31 ± 2.98 points; statistical analysis revealed no significant difference between the two groups in this regard. For the PICC group, pairwise comparisons between CMS scores at one month after catheterization, six months after catheterization, and three months after extubation showed that the score at six months after catheterization was statistically significantly lower than that at one month after catheterization and three months after extubation, respectively. For the TIVADs group, pairwise comparisons between CMS scores at one month after catheterization, six months after catheterization, and three months after extubation showed no statistically significant differences (Tables 3 and 4).

**Table 3:** Comparison of the Two Groups in terms of Constant-Murley Shoulder Function Scores for Catheterization Side

	PICC	TIVADs	T value	*P*
One month after catheterization	94.00 ± 2.47	94.37 ± 3.01	−0.564	0.575
Six months after catheterization	90.71 ± 2.81	94.20 ± 3.20	−5.007	0.034 < 0.05
Three months after extubation	93.66 ± 2.27	94.31 ± 2.98	−1.037	0.303

PICC: peripherally inserted central catheter; TIVADs: implantable venous access device

**Table 4:** Pairwise Comparison across the Two Groups of Constant-Murley Shoulder Function Scores for the Catheterization Side

	One month after catheterization	Six months after catheterization	Three months after extubation	F value	*P*
PICC	94.00 ± 2.47	90.71 ± 2.81	93.66 ± 2.27	17.835	0.000
TIVADs	94.37 ± 3.01	94.20 ± 3.20	94.31 ± 2.98	0.030	0.971

PICC: peripherally inserted central catheter; TIVADs: implantable venous access device

### Displacement of the position of the catheter end after 180° abduction of the catheterization-side shoulder joint from the neutral position

**Fig. 1:**
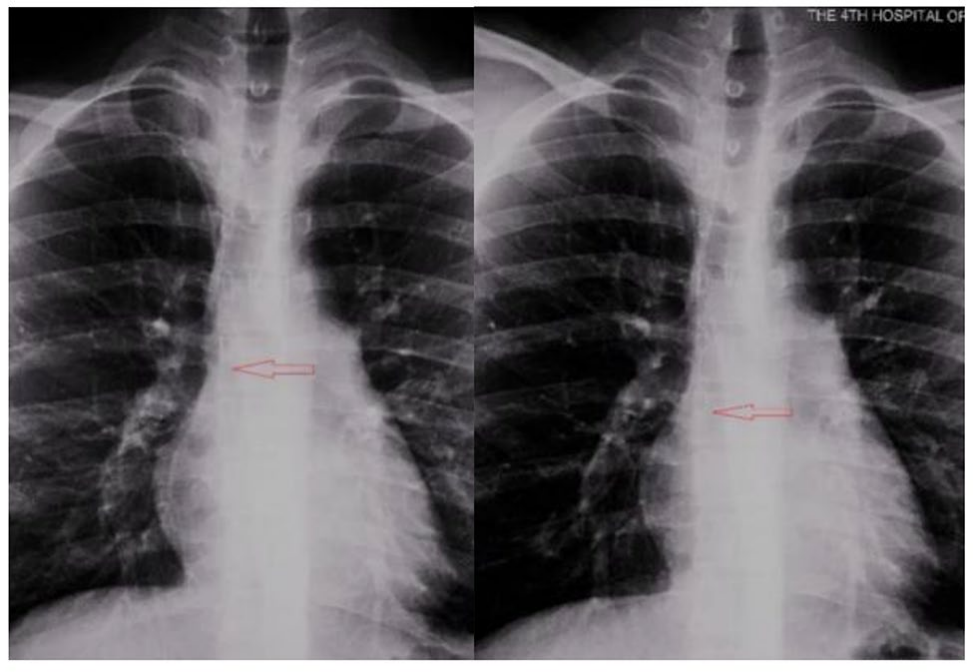
Position Movement due to Catheterization-Side Shoulder Joint’s 180° Abduction from Neutral Position *Note.* The movement is marked by a red arrow

**Fig. 2:**
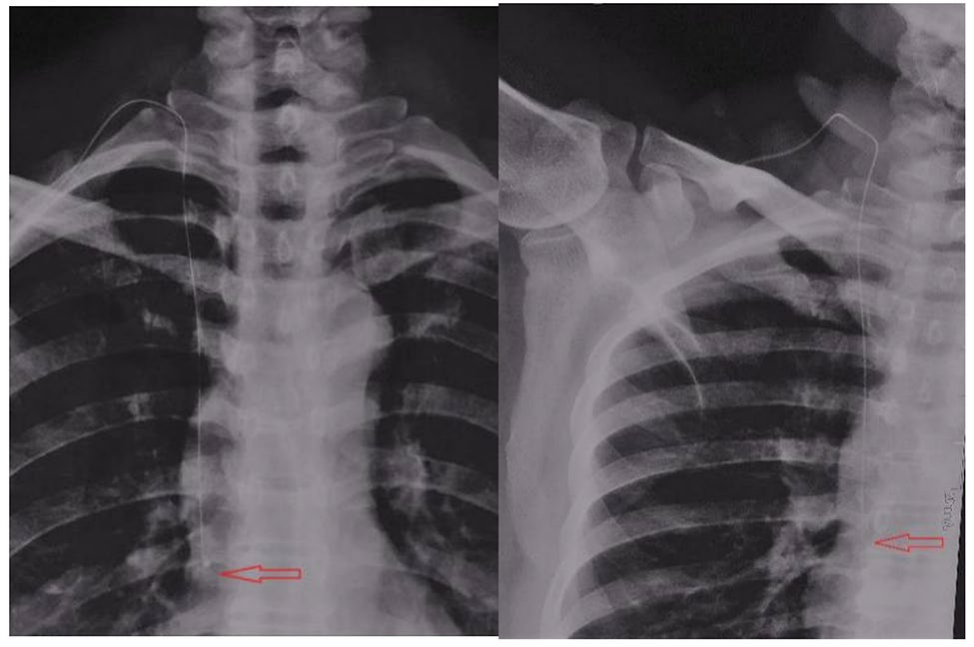
Port Catheter’s End-Position Movement due to Catheterization-Side Shoulder Joint’s 180° Abduction from Neutral Position (*Note.* The movement is marked by a red arrow.)

In both groups, the end of the catheter tended to move to the right atrium. The displacement distance was 1.67 ± 0.43 cm in the PICC group and 0.45 ± 0.15 cm in the TIVADs group, and statistical analysis revealed a significant difference between the two groups in this regard (Table 5; Figs. 1 and 2).

**Table 5:** Comparing Both Groups vis-à-vis Catheter’s End-Position after Catheterization-Side Shoulder Joint’s 180° Abduction from Neutral Position

	PICC (n = 65)	TIVADs (n = 65)	*T*	*P*
Displacement distance of catheter end (cm)	1.67 ± 0.43 cm	0.45 ± 0.15 cm	13.199	0.000

PICC: peripherally inserted central catheter; TIVADs: implantable venous access device

### Catheter complications

**Fig. 3:**
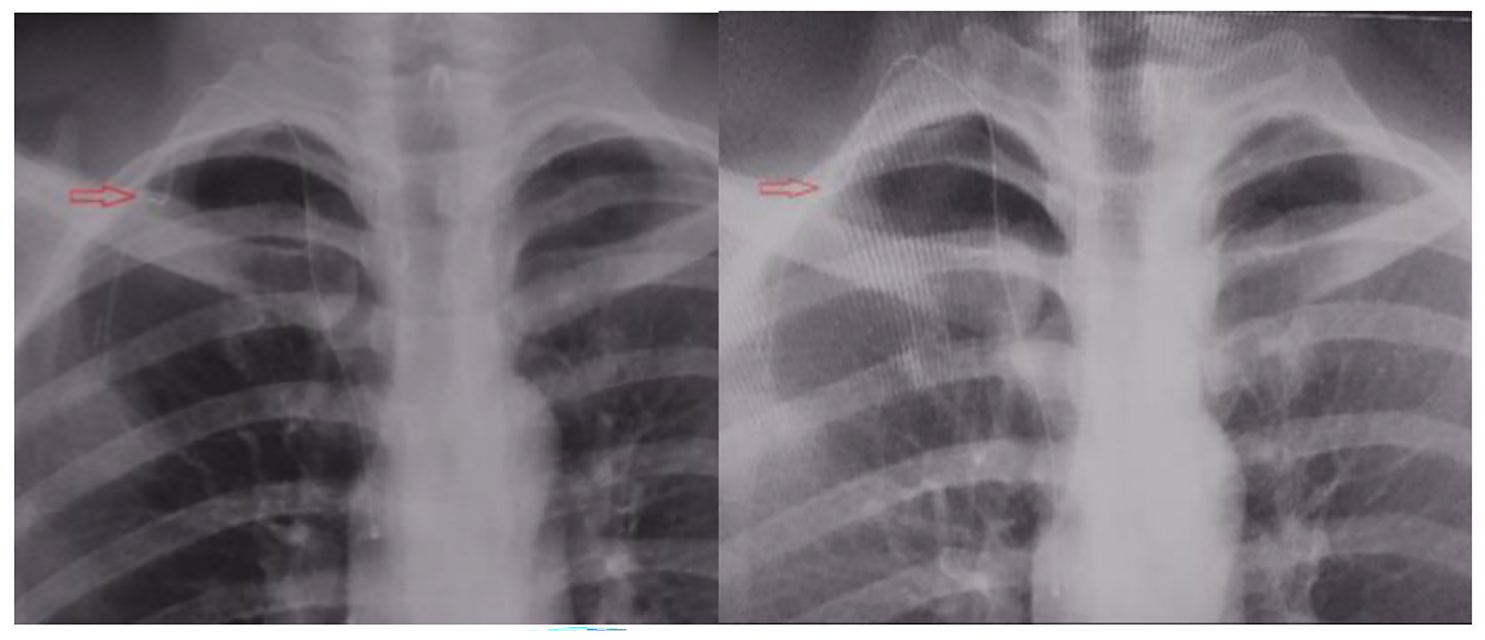
Before and After Images of Catheter Relocation for Port Displacement and Folding *(Note.* The change is marked by a red arrow.*)*

There were 16 cases (24.62%) of catheter related complications in PICC group, including 14 cases (21.54%) of infusion inflammation and two case (3.08%) of catheter prolapse; There were four cases (6.15%) of catheter related complications in TIVADs group, including 2 case (3.08%) of catheter displacement, which was addressed through relocation, and 2 case (3.08%) of intra-catheter thrombosis, which was addressed through thrombolytic therapy. The risk of catheter related complications in PICC group was 4.88 times that in TIVADs group, and the difference was statistically significant (χ^2^ = 4.200, P = 0.04). There were no cases of catheter infection or catheter blockage (Table 6; Fig. 3).

**Table 6:** Comparison of the Two Groups in terms of Catheter Complications

Group	Soft tissue sarcoma (n = 30)	Bone tumor (n = 35)	Total	OR	χ^2^	*P*
PICC (n = 65)	Infusion inflammation (n = 8, 12.31%)	Infusion inflammation (n = 6, 9.23%) Prolapse (2, 3.08%)	N = 16 (24.62%)	4.88	4.200	0.04
TIVADs (n = 65)	Catheter displacement (n = 2, 3.08%)	Thrombosis (2, 3.08%)	N = 4 (6.15%)			

PICC: peripherally inserted central catheter; TIVADs: totally implantable venous access device

## Discussion

Central venous access is essential for preoperative neoadjuvant chemotherapy and postoperative long cycle chemotherapy for patients with malignant bone and soft tissue tumors of lower extremities. Intravenous Infusion Harbor is a fully enclosed, central infusion access that addresses some of the problems caused by PICC use in chemotherapy patients, such as affecting walker use, increasing pain from repeated piercings, treatment costs, and catheter complications. At present, it has been used in chemotherapy and other treatments for a variety of tumor diseases, bringing great convenience to patients and medical staff, and becoming a “green channel” of chemotherapy for patients with malignant bone and soft tissue tumors of lower extremities.

***Application of TIVADs helps to maintain good shoulder function***.

*-----displacement range of the catheter tip is less affected (compared to PICC) by shoulder adduction and abduction*.

The majority of patients with malignant bone and soft tissue tumors of the lower extremities also experience lower-limb movement disorders. Some amputees require walkers or axillary canes to assist their daily life activities, meaning, for such patients, upper-limb function is highly important for the performance of such activities. Thus, such patients have high requirements in terms of shoulder joint function.

The effect of limb movement, especially the movement of the catheterization-side shoulder joint, on the position of the central catheter tip remains debated. Most studies have found that for patients with PICC, in a 90° abduction of the shoulder joint, the catheter tip tends to move towards the right atrium; meanwhile, when the shoulder joint goes past a 90° abduction, the catheter tip tends to move towards the superior vena cava. Finally, if the abduction is continued and reaches 110–180°, the catheter tip moves back towards the right atrium [20, 21]. In our study, we found that for patients with PICC, when the catheterization-side shoulder joint was abducted 180°, the catheter tended to move towards the right atrium, with an average displacement distance of > 1.5 cm; meanwhile, for patients implanted with venous ports in the internal jugular vein, the location of the catheter tip was less affected by shoulder joint adduction and abduction, showing an average displacement distance of < 0.5 cm (towards the right atrium). There was a statistically significant difference between the two groups in this regard. Thus, when providing guidance in regard to functional exercise movements to patients who have received PICC, the patients should be informed that they should minimize 180° abduction of the catheterization-side shoulder joint; this is in order to prevent arrhythmia and even endocardial necrosis and perforation caused by downward displacement of the catheter [22].

In our study, we found no significant difference between the PICC group and the TIVADs group regarding CMS score for the catheterization-side shoulder at one-month post-catheterization. However, at six months post-catheterization, the patients in the PICC group scored significantly lower than the patients in the TIVADs group; this may have been caused by limitations regarding active movement of the catheterization-side shoulder joint after catheterization. Most medical staff do not provide sufficient post-catheterization guidance to patients regarding functional exercise for the shoulder joint [23]. Additionally, patients with PICC showed shoulder-joint adhesion on the catheterization side at six months after catheterization, meaning they had a small range of motion in the shoulder joint in question and, thus, a lower CMS score on the catheterization side. This adhesion may have been caused by the adoption of an abnormal position due to unusual shoulder joint activity or their own fear of catheter prolapse. At three months after extubation, there was no statistically significant difference between the two groups regarding CMS scores. Concurrently, focusing solely on the PICC group, there was no significant difference in CMS scores for the catheterization-side shoulder between one month after catheterization and three months after extubation. For the patients in the TIVADs group, there was no significant difference in CMS score for the catheterization side across the time points of one month after implantation, six months after implantation, and three months after extubation, respectively, which further confirmed the superiority of venous port implantation in regard to optimizing shoulder joint function. we collected CMS data at 1 month, 3 months, and 6 months, and there was not a recollection bias.

Thus, although, for patients who receive PICC, activity of the catheterization-side shoulder joint will affect the position of the catheter tip, medical staff should guide these patients to perform upper limb functional exercise within a safe range (180° abduction of shoulder should be avoided); this guidance should be tailored to the actual situation of each patient, and should allow the patients to maintain good shoulder joint function and good quality of life throughout their treatment.

### TIVADs is more advantageous than PICC in terms of catheter indwelling time

Chemotherapy for bone tumors and soft tissue sarcomas can be characterized by long cycles and high dosages, this means higher requirements in regard to infusion access. In the present study, patients with PICC were extubated within two weeks of their completion of chemotherapy (the average extubation time for patients with soft tissue sarcomas was 233.35 ± 10.43 days, and the average extubation time for patients with malignant bone tumors was 349.54 ± 15.08 days). For patients in the TIVADs group, their ports were removed if no abnormality was found at a re-examination conducted three months after completion of chemotherapy (the average extubation time for patients with soft tissue sarcomas was 323.90 ± 7.77 days, and the average extubation time for patients with malignant bone tumors was 450.13 ± 13.56 days). Thus, TIVADs have more advantages than PICC regarding the long-term retention of catheters.

### Incidence of TIVADs-related complications is relatively low

The overall incidence of complications in the TIVADs group was significantly lower than that in the PICC group. The risk of catheter related complications in PICC group was 4.88 times that in TIVADs group, the difference was statistically significant (χ^2^ = 4.200,P = 0.04).Therefore, when compared with PICC, TIVADs has the advantages of a longer possible indwelling time and a lower risk of chemotherapy-related complications for both soft tissue sarcomas and bone tumors.

In previous studies of patients receiving long-term chemotherapy, when compared with PICC venous port showed the advantages of a low complication rate and high patient satisfaction [24]. Although PICC catheterization can have a low risk of causing damage to surrounding vital tissues and organs, the overall incidence of complications is nevertheless high; this is probably caused by: long-distance catheterization, large variations in peripheral infusion approaches, exposure of the catheterization end, and the small distance between the puncture site and the elbow joint (which has a large range of motion). Further, patients with PICC require weekly catheter care, and some patients have low compliance in this regard; this can also increase the risk of PICC complications, especially catheter-related infections, and thrombosis (particularly among patients with malignant tumors) [25]. Such complications may be mainly attributed to such patients’ hypercoagulable state and reduced immune function. In contrast, the incidence of complications in patients using ports is relatively low, generally < 10%; early complications mainly include mis-penetration into the artery, pneumothorax, and arrhythmias caused by excessively deep catheterization, while late complications mainly include catheter displacement, fracture, infection, and venous thrombosis [26–29]. Among all complications, TIVADs infection and catheter-related infection are the most common [30]. However, there is controversy regarding the use of antibiotics to prevent port device infection. Johnson et al. [31] conducted a meta-analysis of four studies that reviewed antibiotic prophylaxis in port placement; these four studies featured a total of 2,154 patients with port implantation, of whom 360 (16.7%) used antibiotics and 1,794 (83.3%) did not; overall, 27 (1.25%) of these patients developed infection. Of the infected patients, five were receiving antibiotic prophylaxis (1.39%) and 22 were not receiving antibiotic prophylaxis (1.23%). Consequent statistical analysis revealed that prophylactic use of antibiotics does not reduce the incidence of infection after port implantation. Additionally, the use of TIVADs may lead to a higher incidence of complications during the application of targeted drug therapy for patients with malignant tumors; the most common complications in this regard are infection and dehiscence of the port incision [32]. Therefore, to reduce the risk of related complications, before implementing targeted therapy for patients with bone tumors and soft tissue sarcomas who have received conventional chemotherapy it is recommended that the port be removed.

At present, the primary implantation sites for venous ports include the internal jugular vein, the subclavian vein, the basilic vein, and the femoral vein. However, implantation through the subclavian vein may lead to “pinch-off” syndrome [33], which is when the catheter enters the subclavian vein through the gap between the first rib and the clavicle; it is then squeezed by the first rib and the clavicle, resulting in stenosis or clamping, which can cause catheter injury or fracture in severe cases. In addition, implantation through the femoral vein may lead to deep venous thrombosis of the lower extremities [34]. Thus, these two sites are limited in terms of their clinical application, and implantation through the internal jugular vein is the most common approach. In our study, the patients mainly had bone and soft tissue tumors of the lower extremities, were relatively young, and had movement disorders of the lower extremities. A small number of amputees needed to walk and live with the help of walkers or axillary canes, meaning they had high requirements for upper limb function. Therefore, internal jugular vein implantation was the most common approach.

### Study Limitations

Despite its strengths, this study has some shortcomings. Primarily, the number of cases examined was relatively small, bias could not be avoided in the statistical analysis, and the effect of patient position was not considered when examining displacement of the location of the catheter end. These shortcomings must be addressed in future clinical work.

### Implications for nursing

In clinical work, patients who have received chemotherapy for bone and soft tissue tumors of the lower extremities often have lower-limb dysfunction. Some patients who have undergone amputation need to use walkers or axillary canes to perform daily activities. They also have high requirements for upper-limb and shoulder-joint function. A PICC must be placed in the upper limb and, thus, has a certain impact on patients’ upper limb activities, such as their ability to use walking aids or axillary canes; this, in turn, can affect the patients’ engagement in functional exercise and quality of life. Further, the use of walking aids or axillary canes can increase the risk of PICC tube blockage and other related complications [35].The port approach solves the above issues; the patients can use walkers or axillary canes flexibly, which improves their quality of life, and TIVADs also has the advantages of long indwelling time and low risk of complications. Thus, health-care professionals should carefully consider the optimal chemotherapy pathways for patients with lower-extremities bone and soft tissue tumors. Additionally, this study can represent a reference for nurses who are guiding patients who have received a venous port regarding the range of motion they can apply in their upper extremities and shoulder joints during daily activities.

## Conclusions

For patients with bone and soft tissue tumors of the lower extremities, when compared with conventional PICC, use of totally implantable venous access device for long-term chemotherapy has the advantages of longer catheter indwelling time, lower risk of complications, better shoulder joint function, and less risk of catheter end displacement due to shoulder joint activity. Thus, this approach is worthy of promotion in clinical work, and TIVADs should be prioritized for patients with osteosarcoma who are undergoing chemotherapy.

## Data Availability

The datasets generated and analyzed during the current study are not publicly available due to the nature of this research, participants of this study did not agree for their data to be shared. But raw/anonymized data is available from the corresponding author on reasonable request.

## Abbreviations

CMS: Constant-Murley Score
ECOG-PSR: Eastern Cooperative Oncology Group - Performance Status Rating
PICC: Peripherally inserted central catheter
TIVADs: Totally implantable venous access device
